# Nonclinical pharmacodynamics of boron neutron capture therapy using direct intratumoral administration of a folate receptor targeting novel boron carrier

**DOI:** 10.1093/noajnl/vdae062

**Published:** 2024-04-22

**Authors:** Kohei Tsujino, Hideki Kashiwagi, Kai Nishimura, Yoshiki Fujikawa, Ryo Kayama, Yusuke Fukuo, Ryo Hiramatsu, Naosuke Nonoguchi, Takushi Takata, Hiroki Tanaka, Minoru Suzuki, Naonori Hu, Koji Ono, Masahiko Wanibuchi, Kei Nakai, Hiroyuki Nakamura, Shinji Kawabata

**Affiliations:** Department of Neurosurgery, Osaka Medical and Pharmaceutical University, Takatsuki City, Japan; Department of Neurosurgery, Osaka Medical and Pharmaceutical University, Takatsuki City, Japan; Laboratory for Chemistry and Life Science, Institute of Innovative Research, Tokyo Institute of Technology, Yokohama, Japan; Department of Neurosurgery, Osaka Medical and Pharmaceutical University, Takatsuki City, Japan; Department of Neurosurgery, Osaka Medical and Pharmaceutical University, Takatsuki City, Japan; Department of Neurosurgery, Osaka Medical and Pharmaceutical University, Takatsuki City, Japan; Department of Neurosurgery, Osaka Medical and Pharmaceutical University, Takatsuki City, Japan; Department of Neurosurgery, Osaka Medical and Pharmaceutical University, Takatsuki City, Japan; Institute for Integrated Radiation and Nuclear Science, Kyoto University, Sennan-gun, Japan; Institute for Integrated Radiation and Nuclear Science, Kyoto University, Sennan-gun, Japan; Institute for Integrated Radiation and Nuclear Science, Kyoto University, Sennan-gun, Japan; Kansai BNCT Medical Center, Osaka Medical and Pharmaceutical University, Takatsuki City, Japan; Kansai BNCT Medical Center, Osaka Medical and Pharmaceutical University, Takatsuki City, Japan; Department of Neurosurgery, Osaka Medical and Pharmaceutical University, Takatsuki City, Japan; Department of Radiation Oncology, Faculty of Medicine, University of Tsukuba, Tsukuba, Japan; Laboratory for Chemistry and Life Science, Institute of Innovative Research, Tokyo Institute of Technology, Yokohama, Japan; Department of Neurosurgery, Osaka Medical and Pharmaceutical University, Takatsuki City, Japan

**Keywords:** boron neutron capture therapy, convection-enhanced delivery, folate receptor, glioblastoma, malignant glioma

## Abstract

**Background:**

Boron neutron capture therapy (BNCT) is a precise particle radiation therapy known for its unique cellular targeting ability. The development of innovative boron carriers is crucial for the advancement of BNCT technologies. Our previous study demonstrated the potential of PBC-IP administered via convection-enhanced delivery (CED) in an F98 rat glioma model. This approach significantly extended rat survival in neutron irradiation experiments, with half achieving long-term survival, akin to a cure, in a rat brain tumor model. Our commitment to clinical applicability has spurred additional nonclinical pharmacodynamic research, including an investigation into the effects of cannula position and the time elapsed post-CED administration.

**Methods:**

In comprehensive in vivo experiments conducted on an F98 rat brain tumor model, we meticulously examined the boron distribution and neutron irradiation experiments at various sites and multiple time intervals following CED administration.

**Results:**

The PBC-IP showed substantial efficacy for BNCT, revealing minimal differences in tumor boron concentration between central and peripheral CED administration, although a gradual decline in intratumoral boron concentration post-administration was observed. Therapeutic efficacy remained robust, particularly when employing cannula insertion at the tumor margin, compared to central injections. Even delayed neutron irradiation showed notable effectiveness, albeit with a slightly reduced survival period. These findings underscore the robust clinical potential of CED-administered PBC-IP in the treatment of malignant gliomas, offering adaptability across an array of treatment protocols.

**Conclusions:**

This study represents a significant leap forward in the quest to enhance BNCT for the management of malignant gliomas, opening promising avenues for clinical translation.

## Lay Summary

Boron neutron capture therapy (BNCT) is a treatment that uses targeted neutron radiation to kill cancer cells that have absorbed a boron compound. PBC-IP is a new drug that carries boron and it can be delivered directly into brain tumors through a method called convection-enhanced delivery (CED). Prior research has shown that direct delivery of PBC-IP using CED followed by targeted radiation increased the survival of rats with brain tumors. The authors of this study looked to see if where they delivered the PBC-IP and when they gave neutron radiation made a difference in how well the treatment worked. To do this they treated rats with brain tumors by delivering PBC-IP in the middle or edge of the tumor and then gave neutron radiation at different time points. Their results show treatment with delivery of PBC-IP to the center or the edge of the tumor, and radiation for up to 24 hours after delivery of PBC-IP were all similarly effective.

**Key Points**

1. PBC-IP shows promise for BNCT, holding potential in treating malignant gliomas effectively.
2. Convection-enhanced delivery administration of PBC-IP in BNCT presents a promising therapeutic approach.
3. Preclinical trials ready to unlock the full potential of PBC-IP in glioma treatment.

**Importance of the Study**

This study used a rat brain tumor model to investigate the potential of a novel folate receptor-targeted boron carrier combined with convection-enhanced delivery (CED) as a treatment strategy for BNCT in malignant gliomas. Notably, it addresses the practical challenges associated with variations in the cannula insertion positions, a concern that is directly relevant to patient treatment. In addition, we explored the optimal timeframe for effective neutron irradiation post-administration. BNCT comprises a 2-step treatment involving boron agent administration followed by neutron irradiation and is ideally suited for CED administration. Nontoxic boron compounds allow flexibility in timing to optimize drug accumulation in tumors and clearance from normal tissues. BNCT, utilizing CED administration of PBC-IP with its tumor-selective folate receptor targeting and high boron retention in the tumor, has emerged as a promising, adaptable treatment option for malignant gliomas with potential for future clinical applications.

Boron neutron capture therapy (BNCT), which selectively destroys tumor cells at the cellular level, has attracted attention as a potential treatment option for malignant gliomas. Malignant gliomas, particularly glioblastomas, have a poor prognosis. Despite conventional treatments such as surgical resection and postoperative chemoradiotherapy, achieving a complete cure remains challenging owing to the widespread infiltration of tumor cells into the surrounding normal brain tissue.^1^ Recent attempts to introduce novel treatments for glioblastoma have yielded limited success.^2,3^ Consequently, there is an urgent need to develop effective treatments for glioblastoma. In the clinical application of BNCT, tumor cells are targeted by boron compounds, followed by precise neutron irradiation. The high-energy particle-beam-generated nuclear capture reaction of boron atoms (B^10^) with thermal neutron beams has cell-level accuracy, with an effective diameter of approximately 10 μm per cell. This ensured that the cell-killing effect of neutron irradiation was limited to tumor cells that successfully acquired boron-10. In clinical BNCT, boronophenylalanine (BPA) is selectively taken up by tumor cells through the highly expressed L-type amino acid transporter-1 (LAT-1).^4^ BPA is not limited to gliomas but has also been employed in BNCT for head and neck cancers.^5^ Although the clinical effectiveness of BPA-based BNCT using reactor and accelerator neutron sources has been previously established,^6–13^ there is a pressing need to develop novel boron carriers with a mechanism distinct from that of BPA. This is crucial for broadening the spectrum of conditions treatable with BNCT and enhancing treatment efficiency. Our preliminary research showed that BNCT with pteroyl-*closo*-dodecaborate-conjugated 4-(p-iodophenyl) butyric acid (PBC-IP), which contains ten B^10^ atoms (*closo*-dodecaborate), maybe a promising treatment for malignant gliomas. PBC-IP features folate receptor α (FRα) targeting,^14^ enabling higher boron concentration within tumor tissues, such as malignant gliomas, where FRα is significantly overexpressed compared to normal tissues.^15–18^ This innovative approach offers exciting potential for expanding the application of BNCT and improving its efficacy.

A U87 MG human glioma cell subcutaneous xenograft mouse model demonstrated substantially superior tumor growth inhibition when BNCT was conducted following intravenous administration of PBC-IP compared to BNCT with BPA intravenous.^14^ When applied to brain tumors, the PBC-IP was administered via convection-enhanced delivery (CED) in an F98 rat glioma model because of its inability to cross the blood-brain barrier. BNCT using the PBC-IP CED exhibited remarkable results in our pilot study, significantly extending the survival of rats with malignant gliomas. In particular, a significant difference was observed between BNCT PBC-IP CED and BNCT with intravenous BPA, emphasizing the therapeutic potential of CED-administered PBC-IP. Remarkably, half of the rats in the BNCT PBC-IP CED group achieved long-term survival, resembling a cure.^14^ These findings strongly suggest that PBC-IP holds great promise as a boron carrier in BNCT for the effective treatment of malignant gliomas.

This compelling outcome motivated us to pursue further experimental investigations to facilitate clinical application. This study investigated how the effectiveness of treatment varies depending on the location of the CED catheter and the time since its administration. The goal was to establish a protocol for the next phase of the clinical trials. This study is divided into 2 main parts. First, we examined the extent to which the effect of the treatment decreased when the catheter was positioned at the periphery of the tumor core. Second, we explored the acceptable timeframe between the end of administration and the commencement of treatment.

## Material and Methods

### Boron Carriers and Synthesis

In this study, 2 boron carriers, PBC-IP and BPA, were used. PBC-IP, synthesized following our previous investigation, has the chemical structure detailed in our publication by Nishimura et al.^14^ PBC-IP had a molecular weight (MW) of 1145.96, and a boron-10 enrichment rate of 99.8%. L-4-BPA was purchased from Interpharma Praha (Prague, Czech Republic) and transformed into a fructose complex.^19^ BPA used in this study was boron-10-enriched.

### Cell Lines and Culture Conditions for Glioma Cells

The F98 and C6 rat glioma cell lines were used in this study. F98 cells were provided by Dr. Rolf Barth (The Ohio State University, Columbus, OH, USA), and C6 cells were purchased from the Japanese Collection of Research Bioresources Cell bank at the National Institute of Biomedical Innovation (Osaka, Japan). Cells were maintained at 37°C in a 5% CO2 atmosphere, cultured in Dulbecco’s Modified Eagle’s medium (DMEM) supplemented with 10% fetal bovine serum, penicillin, streptomycin, and amphotericin B. All cell culture materials were obtained from Gibco Invitrogen (Grand Island, NY, USA).

### Cell Culture and Boron Exposure Experiments in Glioma Cells

Cultured for 3 days on 100 mm dishes (Becton, Dickson, and Company, Franklin Lakes, NJ, USA), 5.0 × 10^5^ F98 or C6 rat glioma cells were exposed to BPA or PBC-IP at 5 μg B/mL for 3, 6, and 24 hours. In the clearance phase, the cells were incubated for 24 hours, followed by switching to a boron-free medium for 1, 3, 6, or 24 hours. Three dishes were prepared per group for each duration. After washing with 4% phosphate-buffered saline (PBS), cells were detached using trypsin-ethylenediamine tetraacetic acid solution (2 mL), collected, and counted after centrifugation twice (at 200 × *g* for 5 minutes). The cell pellets were digested in 1 N nitric acid solution (Wako Pure Chemical Industries, Osaka, Japan) overnight. Boron content was quantified using inductively coupled plasma atomic emission spectroscopy (ICP-AES; iCAP6300 emission spectrometer, Hitachi, Tokyo, Japan), expressed as μg boron (B)/10^9^ cells.

### Colony-Forming Assay for Assessing Cell-Killing Effects in BNCT

The cell-killing effects of BPA and PBC-IP in BNCT were assessed using a colony-forming assay. F98 and C6 rat glioma cells were divided into neutron-only (exposed to boron-free medium), BPA, and PBC-IP groups. These cells were cultured in 150 cm^2^ flasks, incubated with BPA or PBC-IP at 5 μg B/mL for 24 hours, and then exposed to neutron irradiation for 10, 20, and 30 minutes at a reactor power of 1 MW with a neutron flux of 1.0 × 10^9^ neutrons/cm^2^/s at KURNS. Following neutron irradiation, the cells were seeded into 60 mm dishes (Becton, Dickson and Company), fixed using 90% ethanol, and stained with Giemsa after a 7-day incubation. Colonies consisting of more than 50 cells were counted, and the counts were normalized to those of the control group to calculate the SF (Survival fraction). The relative biological effectiveness and compound biological effectiveness (CBE) for each boron carrier were determined at SF = 0.1 using the linear-quadratic model obtained from X-ray irradiation (M-150WE; SOFTEX).^20^

### F98 Rat Glioma Models

In all the in vivo studies, F98 rat glioma cells were used because of their ability to replicate human malignant gliomas, particularly during intracerebral implantation. These cells exhibit highly infiltrative growth patterns and low immunogenicity, making them valuable models for the evaluation of therapeutic agents.^21,22^ Male Fischer rats (8 weeks old, weighing 150–200 g; Japan SLC, Shizuoka, Japan) were anesthetized by intraperitoneal injection of a mixture of medetomidine (ZENOAQ, Fukushima, Japan; 0.4 mg/kg), midazolam (SANDOZ, Yamagata, Japan; 2.0 mg/kg), and butorphanol (Meiji Seika, Tokyo, Japan; 5.0 mg/kg). Their heads were fixed using a stereotactic frame (IMPACT-1000C connected to Legato 130; MUROMACHI KIKAI Co., Ltd.). For in vivo experiments, 10^3^ F98 rat glioma cells, suspended in a 10 μL solution of DMEM containing 1.4% agarose (Wako Pure Chemical Industries), were injected at a rate of 20 μL/min using an automated infusion pump. The burr hole for tumor cell implantation was made 1 mm posterior to the bregma and 4 mm to the right lateral side using an electric high-speed drill.^23–27^ All animal experiments adhered to the guidelines for the care and use of laboratory animals and were approved by the Animal Use Review Board and Ethical Committee of Osaka Medical and Pharmaceutical University (No.21085-A) and the Institute for Integrated Radiation and Nuclear Science, Kyoto University (KURNS; Kumatori, Osaka, Japan; No. 2022-38).

### CED in F98 Rat Glioma Models

An Alzet osmotic pump (model #2001D, Durect) was used to administer drugs to the Fischer rats through CED. A predetermined boron concentration of 200 µL of BPA or PBC-IP was loaded into the pump, and the device was assembled by connecting the infusion cannula to a brain infusion kit 2 (rigid stainless-steel cannula, 5-mm 28 gauge). Following the previously described anesthesia procedure, a skin incision was made on the back of the rat, and the device was implanted subcutaneously. On the head side, a brain infusion kit was inserted into the burr hole and the wound was closed with a 3–0 thread.^23,24^ This Alzet osmotic pump delivers 200 µL of the boron carrier at a rate of 8 μL/h.

### In Vivo Biodistribution of Boron in F98 Rat Glioma Models

When the implanted F98 glioma cells have become a sufficient size, CED administration of BPA (1000 μg B/mL, equivalent to 1.2 mg B/b. w) and PBC-IP (250, 500, or 1000 μg B/mL, corresponding to 0.3, 0.6, or 1.2 mg B/b. w.) began. Three PBC-IP approaches at 500 μg B/mL were employed: catheter insertion into the tumor center (original implantation site), catheter placement at the tumor border (2 mm posterior to implantation site), and contralateral brain catheter insertion (1 mm posterior to bregma, 4 mm to the left lateral side).

The rats were euthanized at specific time intervals (Table 1), and various tissues (including the tumor, ipsilateral brain, contralateral brain, blood, heart, lung, liver, kidney, spleen, skin, muscle, adrenal gland, and intestine samples) were collected. The weight of each organ was recorded. After digestion in 1 N nitric acid, the amount of boron was quantified using ICP-AES. Boron concentrations were expressed as μg boron (B)/g.

**Table 1.** Summary of the Boron Concentrations After Convection-Enhanced Delivery Administration of Each Boron Carrier in the F98 Rat Glioma Models

Boron carrier	Route	Boron concentration (μg B/mL)	Time ^a^ (h)	*n* ^b^	Boron concentrations ± SD (μg B/g) ^c^				Ratios	
					Tumor	Ipsilateral brain	Contralateral brain	Blood	T/Br ^d^	T/Bl ^e^
PBC-IP	CED	250	3	6	18.5 ± 12.5	1.5 ± 2.2	0.3 ± 0.2	0.3 ± 0.1	59.0	64.5
			6	4	12.8 ± 9.1	0.5 ± 0.4	0.2 ± 0.1	0.2 ± 0.1	66.9	71.7
		500	1	5	68.6 ± 14.8	3.6 ± 1.5	0.6 ± 0.3	0.4 ± 0.2	108.6	185.1
			3	6	29.7 ± 6.6	2.4 ± 1.3	0.5 ± 0.1	0.3 ± 0.1	59.5	97.8
			6	5	18.2 ± 3.7	1.9 ± 0.9	0.5 ± 0.2	0.4 ± 0.1	38.3	52.3
			12	3	11.6 ± 4.4	1.3 ± 0.8	0.4 ± 0.2	0.2 ± 0.1	30.2	52.4
			24	3	4.6 ± 3.2	0.9 ± 0.4	0.3 ± 0.2	0.2 ± 0.1	17.0	21.9
			48	3	1.6 ± 0.5	0.3 ± 0.1	0.1 ± 0.0	0.2 ± 0.1	19.3	11.2
		1000	3	4	60.9 ± 17.8	4.7 ± 1.7	0.9 ± 0.4	0.5 ± 0.2	66.5	135.0
			6	3	37.4 ± 25.9	1.9 ± 1.1	0.6 ± 0.6	0.2 ± 0.1	60.9	167.3
BPA	CED	1000	1	3	13.8 ± 1.0	1.4 ± 0.4	0.8 ± 0.3	0.6 ± 0.0	16.8	23.1
			6	4	4.8 ± 2.6	1.2 ± 0.4	0.6 ± 0.1	0.6 ± 0.1	7.7	8.2
			24	4	0.8 ± 0.1	0.4 ± 0.1	0.3 ± 0.0	0.3 ± 0.0	3.0	2.4
			48	5	0.4 ± 0.2	0.2 ± 0.1	0.2 ± 0.1	0.2 ± 0.0	2.3	1.8

^a^Time indicates hours (h) after the termination of CED administration.

^b^Number of Fischer rats.

^c^Boron concentrations ± SD is measured by ICP-AES for the amount of boron of each organ listed in the table, and is expressed as the mean boron values (μg B/ gr: weight of organ) ± standard deviation.

^d^T/Br indicates the tumor to contralateral brain ratio.

^e^T/Bl indicates the tumor to blood ratio.

CED, convection-enhanced delivery.

### In Vivo Neutron Irradiation for F98 Rat Glioma Models

#### Experimental design and therapeutic evaluation.—

In this study, 2 separate in vivo neutron irradiation sessions were conducted at KURNS on various days. Both sessions took place 14 days after the implantation of 10^3^ F98 rat glioma cells, with the administration of PBC-IP at a concentration of 500 μg B/mL, consistent with previous studies. In both neutron irradiation experiments, the rats were anesthetized via intraperitoneal injection. To ensure that the neutron irradiation was concentrated exclusively on the heads, the entire body of the rats was shielded using a plate lined with ^6^LiF ceramic tiles to minimize extraneous exposure. Subsequently, only their heads were irradiated with neutrons at KURNS, where they received 20 minutes of irradiation at a reactor power of 5 MW and a neutron flux of 9.6 × 10^8^ neutrons/cm^2^/s at the Heavy Water Irradiation Facility. Rats were monitored until they reached an endpoint indicating survival. To evaluate the therapeutic effects, Kaplan–Meier survival curves were employed, and the percent increase in life span (%ILS) was calculated using the following formula: (%ILS) = ([median survival times (MST) of each BNCT group—MST of the control group]/ MST of the control group) × 100.

#### CED duration impact experiment.—

Neutron irradiation experiments were conducted to evaluate the treatment time window for PBC-IP as a boron carrier during neutron irradiation after CED administration. A total of 46 F98 rat glioma models were randomly divided into 6 groups: (1) PBS CED administration (Control [PBS CED]), (2) PBC-IP CED administration (Drug only [PBC-IP CED]), (3) neutron irradiation after termination of PBS CED administration (Neutron only [PBS CED]), (4) neutron irradiation at 3 hours after termination of PBC-IP CED administration (BNCT PBC-IP CED 3 hours), (5) neutron irradiation at 6 hours after termination of PBC-IP CED administration (BNCT PBC-IP CED 6 hours), and (6) neutron irradiation at 24 hours after termination of PBC-IP CED administration (BNCT PBC-IP CED 24 hours).

#### Catheter position effect experiment.—

Subsequent neutron irradiation experiments were conducted to evaluate the effect of different catheter tip positions on the therapeutic effect of CEDs. A total of 30 F98 rat glioma models were randomly divided into 4 groups: (1) untreated group (Control [untreated]), (2) neutron irradiation at 3 hours after the termination of PBC-IP CED administration in the contralateral brain of the tumor (BNCT PBC-IP CED contralateral brain), (3) neutron irradiation at 3 hours after the termination of PBC-IP CED administration in the peritumoral brain (BNCT CED tumor [border]), and (4) neutron irradiation at 3 hours after the termination of PBC-IP CED administration in the peritumoral brain in the tumor body (BNCT PBC-IP CED Tumor [center]). The therapeutic effect was assessed by measuring survival time. In this study, the peritumoral brain was defined as a 2 mm margin surrounding the tumor core.

### The Dose Calculations in the Neutron Irradiation

In BNCT, assessing the absorbed and photon-equivalent doses is crucial. The absorbed dose is determined by the following equation: D_B_ + D_N_ + D_H_ + D_γ_. D_B_ represents the boron dose, corresponding to the ^10^B (n,α) ^7^Li neutron reactions, calculated using the equation 7.43 × 10^-14^ (Gy cm^2^/ μg ^10^B/g) × boron concentration (μg ^10^B/g) × thermal neutron fluence (1/cm^2^). D_N_ is the nitrogen dose resulting from the ^14^N (n, p) ^14^C neutron reactions, determined by the equation: 6.78 × 10^-14^ (Gy cm^2^/weight %) × nitrogen concentration (weight %) × thermal neutron fluence (1/ cm^2^). D_H_ accounts for elastic scattering between epithermal or fast neutrons and the hydrogen nucleus and is associated with ^1^H (n,n) ^1^H neutron reactions. D_γ_ represents the γ-ray dose arising from γ-rays emitted during the capture of a thermal neutron by a hydrogen atom and γ-rays from the source itself, corresponding to the ^1^H (n, γ) ^2^H neutron reactions. Photon-equivalent doses were estimated using the equation: D_B_ × CBE + D_N_ × relative biological effectiveness of nitrogen (RBE_N_) + D_H_ × relative biological effectiveness of hydrogen (RBE_H_) + D_γ_.

RBE_N_ and RBE_H_ were adopted to 3.0 according to previous reports.^28^ Calculation of the photon-equivalent dose to the tumor in the F98 rat glioma model followed the same procedure as that outlined in our previous reports.^23^ The CBE of the PBC-IP tumors was determined using in vitro neutron irradiation. The CBE for the normal brain of PBC-IP was 1.35, which is consistent with that of BPA,^8,29^ under the assumption of equal boron-derived biological effectiveness for both BPA and PBC-IP.

### Statistical Analysis

The Student’s *t*-test was used to compare tumor cell boron concentrations in the in vitro uptake experiment, SF (Survival Fraction) in the in vitro neutron irradiation test, and the in vivo biodistribution experiment. For the in vivo neutron irradiation experiments, the log-rank test was used to assess group survival, as visualized by Kaplan–Meier curves. Statistical significance (*P* < .05) was determined using JMP® Pro version 16.2.0. software (SAS).

## Results

### In Vitro Cellular Uptake of Boron in F98 and C6 Glioma Cells

**Figure 1.**
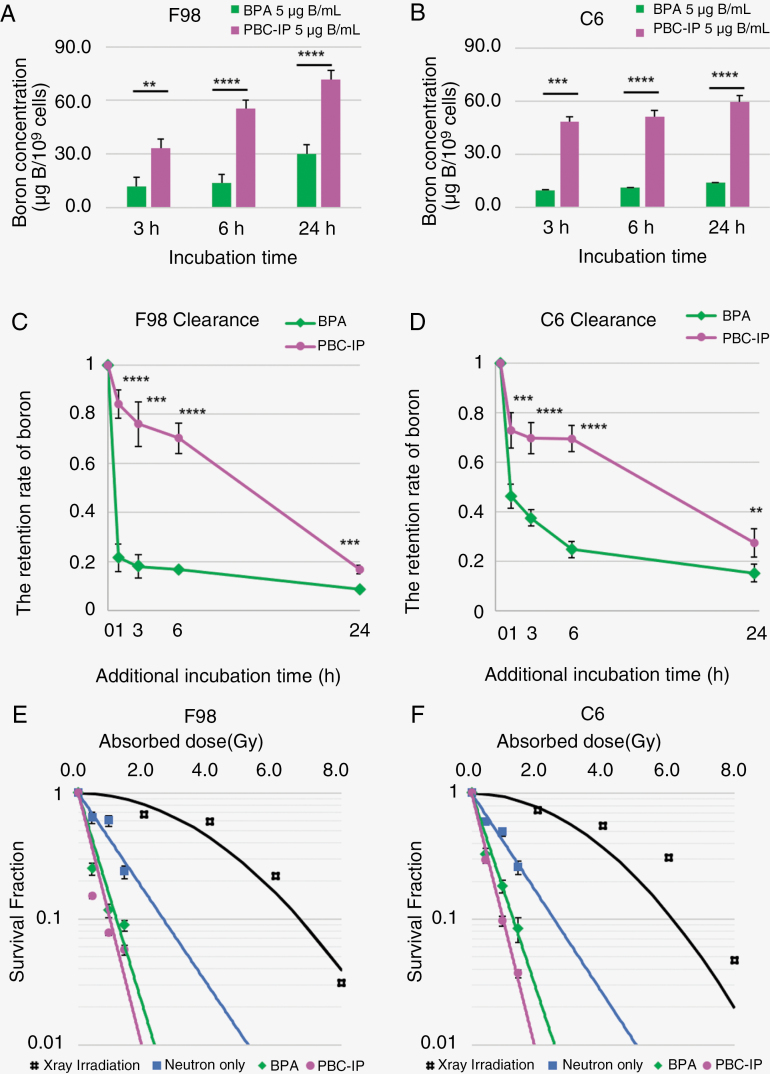
(A,B) These figures show the boron concentrations in F98 and C6 rat glioma cells. The bar indicates the standard deviation. Both cells are exposed to BPA and PBC-IP 5 μg B/mL for 3, 6, and 24 hours. Significant differences are observed between BPA and PBC-IP at all exposure times. (**P < .01 ***P < .001, ****P < .0001). (C,D) These figures show the retention rate of boron in each cell for 1, 3, 6, and 24 hours after the exchange to the boron-free medium from the medium containing BPA or PBC-IP 5 μg B/mL for 24 hours. The bars indicate the standard deviation. Significant differences are observed between the BPA and PBC-IP at all exposure times. (**P < .01 ***P < .001, ****P < .0001). (E,F) These figures show the linear-quadratic (LQ) model for F98 or C6 glioma cells after X-ray irradiation, survival fractions of each group, and approximate lines after neutron irradiation. All results are plotted corresponding to the absorbed dose. The bars indicate the standard error.

The boron concentrations in both BPA and PBC-IP in F98 and C6 rat glioma cells, along with the retention rate of boron, are shown in Figure 1A and B.

From 3 to 24 hours, there was a gradual increase in boron concentrations for both BPA and PBC-IP in both cell lines. Notably, PBC-IP exhibited significantly higher boron concentrations than BPA at all the time points. Moreover, during the clearance phase (from 24 hours to 24 + 24 hours), the boron concentration and retention rate of boron from PBC-IP in both cells were significantly higher than those of BPA.

The retention rate of boron of BPA and PBC-IP after 1, 3, 6, or 24 hours of additional incubation, following the medium change to boron-free medium, were as follows: in F98 glioma cells: 21.6% and 84.1% (1 hour), 18.0% and 76.0% (3 hours), 16.8% and 70.2% (6 hours), 8.6% and 16.7% (24 hours). In C6 glioma cells, 46.3% and 72.9% (1 hour), 37.6% and 69.8% (3 hours), 24.8% and 69.6% (6 hours), 15.2% and 27.5% (24 hours; Figure 1C and D).

### In Vitro Neutron Irradiation for F98 and C6 Glioma Cells

The results of in vitro neutron irradiation of F98 and C6 glioma cells are shown in Figure 1E and F. In the case of neutron irradiation for both cells, the absorbed dose was 0 Gy (0 minute), 0.42 Gy (10 minutes), 0.93 Gy (20 minutes), and 1.41 Gy (30 minutes). In this study, as the absorbed dose increased, all the SFs for both BPA and PBC-IP decreased. Significantly different SFs were observed for BPA and PBC-IP at all irradiation times. In neutron irradiation, the consideration of boron dose, nitrogen dose, hydrogen dose, and γ dose is crucial. The difference between the absorbed dose required to achieve SF = 0.1 in the neutron irradiation group and the absorbed dose required to achieve SF = 0.1 in the photon irradiation group reflects the specific impact of each boron carrier.

The absorbed doses necessary to achieve SF = 0.1 were determined for each group: F98; 2.58 Gy (Neutron only), 1.16 Gy (BPA group), and 0.96 Gy (PBC-IP group), C6; 2.50 Gy (Neutron only), 1.26 Gy (BPA group), and 0.95 Gy (PBC-IP group). In comparison, the absorbed doses needed for SF = 0,1 with X-irradiation were 6.74 Gy (F98), and 6.11 Gy (C6). Neutron beams, characterized by high-energy transfer (LET), beams, necessitate a lower absorbed dose to achieve a comparable biological effect to X-rays. The incorporation of boron enhances these effects, further decreasing the neutron dose equivalent to that of X-ray.

Calculating the CBE of BPA and PBC-IP based on these results yielded the values of 2.65 (BPA) and 3.63 (PBC-IP) for F98, and 2.00 (BPA) and 3.31(PBC-IP) in C6.

### In Vivo Biodistribution of Boron in F98 Rat Glioma Models

**Figure 2.**
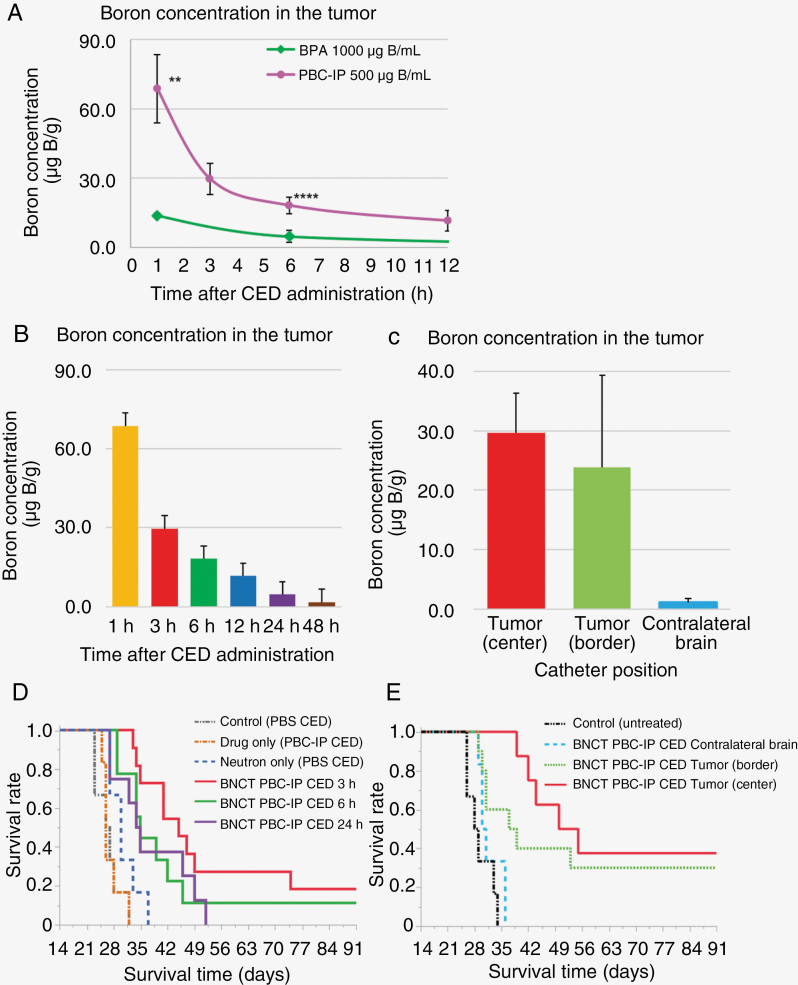
(A) This figure shows the boron concentration in the tumor of BPA 1000 μg B/mL or PBC-IP 500 μg B/mL convection-enhanced delivery (CED) administration. (**P < .01 ***P < .001, ****P < .0001). (B) This figure shows the boron concentration in the tumor from 1 to 48 hours after PBC-IP 500 μg B/mL CED administration. All the bars indicate the standard deviation. (C) This figure shows the boron concentration in the tumor of the each group of the different catheter positions when neutron irradiation was performed. All the bars indicate the standard deviation. (D) This figure shows the Kaplan–Meier curves of the F98 rat glioma models after neutron irradiation for the evaluation of the treatment time window of PBC-IP as a boron carrier in Boron Neutron Capture Therapy (BNCT). In 2 groups (BNCT PBC-IP CED 3 hours and BNCT PBC-IP CED 6 hours), long-term survivors (more than 180 days) are observed. In this figure, the results are shown up to 91 days. (E) This figure shows the Kaplan–Meier curves of the F98 rat glioma models after neutron irradiation for the evaluation of the impact of different catheter tip positions on the therapeutic effect of CED. In 2 groups (BNCT PBC-IP Tumor (border) CED and BNCT PBC-IP CED Tumor (center)), long-term survivors (more than 180 days) are observed. In this figure, the results are shown up to 91 days.

Tables 1 and 2 summarize the in vivo biodistribution of boron in the F98 rat glioma models following CED administration of each boron carrier. For PBC-IP or BPA CED administration, statistically significant differences in boron concentrations were observed between PBC-IP 500 μg B/mL CED and BPA 1000 μg B/mL CED in the tumor, as determined by student’s *t*-test (Figure 2A).

**Table 2.** Summary of Biodistribution of Boron After CED Administration of PBC-IP in the Case of the Different Catheter Positions in F98 Rat Glioma Models

Catheter position ^a^	Time ^b^ (h)	*n* ^c^	Boron concentrations ± SD (μg B/g) ^d^				Ratios	
			Tumor	Ipsilateral brain	Contralateral brain	Blood	T/Br ^e^	T/Bl ^f^
Tumor (border)	3	6	23.8 ± 15.6	7.6 ± 5.6	0.7 ± 0.5	0.3 ± 0.1	34.4	95.3
	6	4	15.4 ± 6.5	6.6 ± 3.5	0.4 ± 0.2	0.3 ± 0.0	34.8	53.9
	24	5	4.5 ± 3.7	3.8 ± 2.5	0.2 ± 0.1	0.3 ± 0.2	19.0	15.0
Contralateral brain	3	3	1.1 ± 0.7	1.3 ± 1.0	40.1 ± 16.8	0.4 ± 0.3	0.03	2.81
	6	3	0.6 ± 0.3	1.3 ± 0.5	10.9 ± 4.2	0.1 ± 0.1	0.05	3.95
	24	4	0.4 ± 0.4	0.6 ± 0.3	5.1 ± 4.9	0.1 ± 0.1	0.09	3.12

^a^The position of the CED catheter shows the name of each defined group corresponding to the position of the CED catheter.

^b^Time indicates hours (h) after the termination of CED administration.

^c^Number of Fischer rats.

^d^Boron concentrations ± SD is measured by ICP-AES for the amount of boron of each organ listed in the table, and is expressed as the mean boron values (μg B/ gram: weight of organ) ± standard deviation.

^e^T/Br indicates the tumor to contralateral brain ratio.

^f^T/Bl indicates the tumor to blood ratio.

CED, convection-enhanced delivery.

The boron concentrations in the tumor, ipsilateral brain, contralateral brain, and blood at 3 h after the termination of CED administration of PBC-IP at 500 µg B/mL were 29.7 ± 6.6, 2.4 ± 1.3, 0.5 ± 0.1, and 0.3 ± 0.1 µg B/g, respectively. After the termination of the PBC-IP CED, the boron concentration in the tumor gradually decreased and remained at high levels for an extended period (Figure 2B). In the comparison of dose escalation based on the boron concentration of the PBC-IP CED administration, a trend toward higher boron concentrations in the tumor with increased PBC-IP dosage was observed. Regarding the different positions of the CED catheter, no statistically significant differences were observed in boron concentrations between PBC-IP CED tumors (center) and PBC-IP CED tumors (border) using Student’s *t*-test (Figure 2C). The boron concentrations in organs (except the tumor, ipsilateral brain, contralateral brain, and blood) were consistently below 1.0 μg B/g in all cases.

### In Vivo Neutron Irradiation for F98 Rat Glioma Models

Figure 2D and E present the Kaplan–Meier curves for 2 distinct in vivo neutron irradiation experiments.

In the first experiment (CED Duration Impact Experiment), the MST for each group was as follows: Control (PBS CED), 26.5 days (95% CI: 23–32 days), Drug only (PBC-IP CED), 26 days (95% CI: 25–32 days), Neutron only (PBS CED), 30 days (95% CI: 27–37 days), BNCT PBC-IP CED 3 hours, 45 days (95% CI: 34–74 days), BNCT PBC-IP CED 6 hours, 35 days (95% CI: 29–46 days), BNCT PBC-IP CED 4 hours, 34.5 days (95% CI: 27–49 days). Statistically significant differences were observed between the control and BNCT groups.

In the second experiment (Catheter Position Effect Experiment), the MST for each group was as follows: Control (untreated), 28.5 days (95% CI: 26–34 days), BNCT PBC-IP CED contralateral brain, 30.5 days (95% CI: 29–36 days), BNCT PBC-IP CED Tumor (border), 38 days (95% CI: 29-NA), and BNCT PBC-IP CED Tumor (center), 52.5 days (95% CI: 39-NA). Statistically significant differences were observed between the control (untreated) group and both the BNCT PBC-IP CED tumors (border) and the BNCT PBC-IP CED tumors (center). Additionally, in both groups of BNCT PBC-IP CED tumors (border) and BNCT PBC-IP CED tumors (center), long-term survivors (more than 180 days after the implantation of F98 cells) were observed (BNCT PBC-IP CED Tumor [border]: 30%; BNCT PBC-IP CED Tumor [center]: 37.5%). The results are presented in Tables 3, 4.

**Table 3.** Summary of the Survival Times, Absorbed Dose, and Estimated Photon-Equivalent Dose of the Brain and Tumor in the F98 Rat Glioma Models in the In Vivo Neutron Irradiation for the Treatment Time Window of PBC-IP as a Boron Carrier in BNCT

Group	*n* ^a^	Survival times (days)			%ILS ^c^	*P*-value ^d^	Absorbed dose ^a^ (Gy)		Photon-equivalent dose ^b^ (Gy-Eq)	
		Mean ± SD	Median	95%CI ^b^			Brain	Tumor	Brain	Tumor
Control (PBS CED)	6	26.5 ± 3.1	26.5	23–32	—	—	0.0	0.0	0.0	0.0
Drug only (PBC-IP CED)	6	27.2 ± 2.3	26	23–32	16.7	.87	0.0	0.0	0.0	0.0
Neutron only (PBS CED)	6	30.7 ± 3.5	30	27–37	13–2	.06	1.0	1.0	2.0	2.0
BNCT PBC-IP CED 3 hours	11	69.0 ± 53.4^*^	45	34–74	69.8	<.0001	1.0	6.7	(2.2)^*^	23.0
BNCT PBC-IP CED 6 hours	9	52.0 ± 45.6^*^	35	29–46	32.1	.0002	1.1	4.3	(2.2)^*^	14.0
BNCT PBC-IP CED 24 hours	8	37.8 ± 9.2	34.5	27–49	30.2	.0054	1.0	1.8	(2.0)^*^	4.8

^a^Number of Fischer rats per group.

^b^CI is confidence interval.

^c^The percent increased life span (%ILS) was defined relative to the mean survival time (MST) of the control (PBS CED) group.

^d^*P*-values were calculated using the log-rank test and compared to the control (PBS CED) group based on the results obtained from the Kaplan–Meier curves in the neutron irradiation for the F98 rat glioma models.

^e^The absorbed dose was calculated as the following equation: D_B_ + D_N_ + D_H_ + D_γ_. All rats in the neutron-only (PBS CED), BNCT PBC-IP CED 3 hours, BNCT PBC-IP CED 6 hours, and BNCT PBC-IP CED 24 hours groups were consistently irradiated for 20 minutes.

^f^Photon-equivalent dose was calculated using the following equation: D_B_ × compound biological effectiveness (CBE) + D_N_ × relative biological effectiveness of nitrogen (RBE_N_) + D_H_ × relative biological effectiveness of hydrogen (RBE_H_) + D_γ_. The CBE for the PBC-IP tumor was obtained from the results of in vitro irradiation. The RBE_N_ and RBE_H_ were adopted as 3.0.

^*^In these groups, long-term survival > 180 days after implantation of 10^3^ F98 rat glioma cells. The survival time (days) of the long-term survivors was 180 days.

^**^In the case of the calculation of the photon-equivalent dose of the normal brain, the CBE for the normal brain of PBC-IP were adopted to 1.35 the same as that of BPA.

BNCT, Boron Neutron Capture Therapy; CED, convection-enhanced delivery.

**Table 4.** Summary of the Survival Times, Absorbed Dose, and Estimated Photon-Equivalent Dose of the Brain and Tumor in the F98 Rat Glioma Models in the In Vivo Neutron Irradiation for the Evaluation of the Impact of Different Catheter Tip Positions on the Therapeutic Effect of CED

Group	*n* ^a^	Survival times (days)			%ILS ^c^	*P*-value ^d^	Absorbed dose ^a^ (Gy)		Photon-equivalent dose ^b^ (Gy-Eq)	
		Mean ± SD	Median	95%CI ^b^			Brain	Tumor	Brain	Tumor
Control (untreated)	6	29.3 ± 3.1	28.5	26-34	—	—	0.0	0.0	0.0	0.0
BNCT PBC-IP CED Contralateral brain	6	32.0 ± 2.9	30.5	29-36	9.1	.18	6.1	1.2	(8.6)^**^	2.3
BNCT PBC-IP CED Tumor (border)	10	79.0 ± 66.4^*^	38	29-NA	169.3	.007	1.2	5.0	(2.1)^**^	16.1
BNCT PBC-IP CED Tumor (center)	8	96.3 ± 65.0^*^	52.5	39-NA	237.1	<.0001	1.1	5.9	(2.1)^**^	19.8

^a^Number of Fischer rats per group.

^b^CI is confidence interval.

^c^The percent increased life span (%ILS) was defined relative to the mean survival time (MST) of the control (untreated) group.

^d^*P*-values were calculated using the log-rank test and compared to the control (untreated) based on the results obtained from the Kaplan–Meier curves in the neutron irradiation for the F98 rat glioma model.

^e^The absorbed dose was calculated as the following equation: D_B_ + D_N_ + D_H_ + D_γ_. All the rats in the BNCT PBC-IP CED Contralateral brain, BNCT PBC-IP CED Tumor (border), and BNCT PBC-IP CED Tumor (center) were consistently irradiated for 20 minutes.

^f^Photon-equivalent dose was calculated using the following equation: D_B_ × compound biological effectiveness (CBE) + D_N_ × relative biological effectiveness of nitrogen (RBE_N_) + D_H_ × relative biological effectiveness of hydrogen (RBE_H_) + D_γ_. The CBE for the PBC-IP tumor was obtained from the results of in vitro irradiation. The RBE_N_ and RBE_H_ were adopted as 3.0.

^*^In these groups, long-term survival > 180 days after implantation of 10^3^ F98 rat glioma cells. The survival time (days) of the long-term survivors was 180 days.

^**^In the case of the calculation of the photon-equivalent dose of the normal brain, the CBE for the normal brain of PBC-IP were adopted to 1.35 the same as that of BPA.

CED, convection-enhanced delivery; NA, not applicable.

### The Estimated Absorbed Dose and Photon-Equivalent Dose in the In Vivo Neutron Irradiation

To calculate the estimated absorbed dose and photon-equivalent dose for the tumor and normal brain, the CBE factors of BPA and PBC-IP obtained from in vitro neutron irradiation were applied. Furthermore, for dose calculation, the boron concentrations in the tumor were based on the mean boron concentrations from the in vivo biodistribution. The dose calculations are summarized in Tables 3 and 4.

## Discussion

This study demonstrates the efficacy of BNCT for malignant gliomas using F98 rat glioma models with the administration of PBC-IP through CED. We conducted experiments to validate the compatibility of the novel boron drug PBC-IP with CED in BNCT. Additionally, we assessed the potential benefits of the PBC-IP by evaluating its in vitro drug properties and conducting 2 in vivo neutron irradiation studies. Within the framework of BNCT, the criteria for an optimal novel boron agent are 3-fold: (1) Maintaining a tumor’s boron concentration at 25–30 μg B/g during neutron irradiation, (2) achieving a tumor/normal brain or blood (T/N or T/Bl) ratio exceeding 3, and (3) ensuring the rapid clearance of the boron agent from normal tissue and blood post-neutron irradiation.^30^

CEDs are direct intracerebral drug delivery systems involving passive and active diffusion.^31^ The local administration of drugs circumvents the challenges posed by the blood-brain barrier, which is a formidable obstacle in delivering drugs to the central nervous system. Furthermore, the ability to administer high drug doses directly to target sites, free from concerns regarding systemic toxicity associated with intravenous administration, is particularly appealing. This attribute of the CED holds great significance in the context of BNCT, where the primary challenge lies in the accumulation of boron within tumor cells. Ongoing clinical trials on malignant gliomas have demonstrated the versatility of CED. Additionally, drugs such as paclitaxel and topotecan, which are typically used in anti-cancer treatments for other cancers, are administered via CED to patients with brain tumors. However, it’s important to note that both drugs possess inherent tumor-killing effects as toxic agents.^32–37^

The advantages of CED administration of boron-carrying drugs in BNCT are not only the reduction of systemic toxic effects or drug delivery to brain tumors unaffected by the blood-brain barrier, as is commonly mentioned. In BNCT, treatment involves 2 sequential steps: Boron carrier administration and neutron irradiation. Assuming that the drug administration itself is nontoxic,^38^ it is possible to plan the required dosage without the risk of toxicity, even when such dosages would be considered infeasible with conventional anti-cancer drugs. Furthermore, as both tumors and normal tissues are gradually cleared over time following the termination of CED administration, neutron irradiation can be scheduled to coincide with a relatively high boron concentration within the tumor, while boron is eliminated from normal tissues. In other words, this occurs when the tumor/normal brain or blood (T/N) ratio is sufficiently high during treatment. The in vivo boron distribution experiment conducted in this study also demonstrated the adequacy of the T/N ratio when PBC-IP was administered via the CED for BNCT. In the case of the intravenous administration of BPA in F98 rat glioma models, the T/N ratio typically remained at approximately 3 or 4.^23–27^

In our in vitro intracellular boron uptake experiments, both cell types exhibited significantly higher boron concentrations in the presence of PBC-IP than in the presence of BPA alone. Moreover, the retention rate of boron following exposure to a medium containing either BPA or PBC-IP was notably higher for PBC-IP than for BPA. To comprehensively assess the utility of this novel boron carrier in BNCT, it is imperative to consider the CBE factor, which is specific to each boron carrier and irradiated tissue. Subsequent in vitro neutron irradiation assays indicated that the cell-killing effect of PBC-IP on BNCT surpassed that of BPA and was sufficient for BNCT (CBE for PBC-IP: 3.63 [F98], and 3.31 [C6]). This distinctive of “long-retention” attribute significantly contributed to the sustained elevation of boron concentrations within the tumor during in vivo neutron irradiation.

The in vivo neutron irradiation experiments in this study were initially conducted to assess the effect of the time elapsed after the termination of CED administration of PBC-IP on therapeutic efficacy. A statistically significant difference was evident between the control group (PBS CED) and all BNCT treatment groups (BNCT PBC-IP CED, 3 hours; BNCT PBC-IP CED, 6 hours; and PBC-IP CED, 24 hours), as determined by the log-rank test (Table 3). Furthermore, it is worth noting that no statistically significant differences were detected between the BNCT PBC-IP CED at 3 hours, BNCT PBC-IP CED at 6 hours, and BCNT PBC-IP CED at 24 hours after the termination of administration. The therapeutic effect of BNCT persisted for at least 24 hours after PBC-IP CED administration, indicating an extended therapeutic time window for BNCT PBC-IP CED. PBC-IP, with its high MW (1145.96) and long retention characteristics, is an attractive boron carrier for CED. Conversely, BPA, with its low MW (209.010), is unsuitable for CED because of its rapid clearance from tumor cells despite its high tumor-accumulating properties.^35^ In fact, the boron concentration within the tumor when BPA is administered via the CED remains insufficient. The attribute of “long-retention” is unique to the novel albumin-based boron carriers previously reported in our studies.^25–27^ These boron carriers are promising for applications involving variable irradiation times for deep-seated, widespread, and multiple lesions. Another crucial observation from the results of in vivo neutron irradiation was that the administration of the drug alone (PBC-IP CED) was insufficient to produce a therapeutic effect; neutron irradiation was an indispensable component for achieving the desired therapeutic outcome.

Drug distribution during PBC-IP CED administration is an essential consideration in clinical applications. It is crucial to recognize that CED can be influenced by tissue properties and the recipient microenvironment, as documented in previous studies.^32,33,35^ Furthermore, variables such as underlying diseases, whether it is an initial or recurrent case, and history of radiotherapy can impact drug distribution. This distribution may differ among the cases. Drug distribution through CED administration plays a pivotal role in determining therapeutic effectiveness. Previous findings indicate that drugs administered via the CED initially accumulate in the necrotic area before diffusing into the peritumoral region, which is attributed to uneven tumor distribution.^39^ The distribution pattern is likely influenced by interstitial fluid flow or intratumoral heterogeneity.^40^ Chen et al. emphasized the importance of identifying optimal catheter positions to achieve uniform drug distribution in the brain.^41^ In clinical trials, inadequate drug distribution, even for a potent drug, can compromise efficacy endpoints, necessitating comprehensive consideration.^40^ Additionally, catheter geometry and administration rate, factors affecting drug distribution, and lack of standardized protocols.^33,42^ In this study, we investigated the impact of varying catheter implantation sites during CED administration on boron concentration within the tumor and its consequent treatment implications. The prior analysis of the in vivo distribution results revealed no significant differences in boron concentrations within the tumors between the 2 groups, namely “CED tumors (center)” and “CED tumors (border).” As expected, the boron concentration in the ipsilateral brain was slightly higher for the “CED Tumor (border)” group compared to the “CED Tumor (center)” group, but this variance remained within an acceptable range.

In an in vivo neutron irradiation experiment aimed at exploring variations in therapeutic efficacy associated with differences in catheter tip positions, both groups, “BNCT PBC-IP CED Tumor (center)” and “BNCT PBC-IP CED Tumor (border),” showed significantly prolonged survival in comparison to the Control (untreated) group. Notably, there was no statistically significant difference between the 2 groups, as confirmed by the log-rank test (“Tumor (center)” vs. “Tumor (border)”; *P* = .3495). Moreover, a notable proportion of patients achieved long-term survival, equivalent to a cure, extending beyond 180 days following the implantation of tumor cells. This suggests that when administering non-cytotoxic boron agents via the CED, it is feasible to achieve doses beyond what is strictly necessary. As long as the agent maintains tumor selectivity, its antitumor effect may not be significantly influenced by histological features. CED, which employs a 2wo-step approach, is a compatible and beneficial approach for administering boron drugs in BNCT. Furthermore, the in vivo biodistribution experiment substantiated a clear and direct correlation; the higher the concentration of PBC-IP administered, the greater the resulting boron concentration within the tumor. As previously discussed, a viable approach to address the issue of drug distribution involves augmenting both the boron concentration and the administered dosage.

The optimization of CED as a delivery method for BNCT in treating brain tumors could lead to improved treatment outcomes, particularly for agents like BPA that are characterized by rapid clearance from tumor tissues. The concept of maintaining continuous CED during irradiation presents a promising avenue for enhancing the efficacy of such treatments. This strategy holds potential not only for compounds with long retention, such as PBC-IP, but also highlights the synergistic potential of combining CED with BNCT. The feasibility and benefits of this approach warrant further investigation.

In a previously published study by Nishimura et al., which discussed the synthesis and drug properties of PBC-IP, it was reported that PBC-IP was widely distributed in the cytoplasm.^14^ In our previous studies, cellular boron concentrations in PBC with folate receptor targeting and BC-IP with albumin ligands were not notably high.^24,27,43^ Although the precise mechanism underlying the cellular uptake of PBC-IP remains uncertain, it is conceivable that the concurrent presence of both albumin ligands and folate receptor targeting may influence their cellular distribution. Considering the greater therapeutic effect demonstrated in the present study, the improved subcellular localization of PBC-IP may have contributed to the microscopic cell-killing effect of BNCT. These results are informative for nonclinical trials, although further basic research is needed to explore the microscopic cellular distribution and therapeutic efficacy of neutron capture therapy more comprehensively.

## Conclusion

The novel boron carrier, “PBC-IP,” has emerged as a highly promising agent for BNCT, demonstrating significant potential in the management of malignant gliomas. The application of BNCT with PBC-IP via CED is a promising treatment avenue. Further investigation and preclinical trials are warranted to fully explore this therapeutic approach.

## Data Availability

The datasets analyzed in the current study are available from the corresponding author upon reasonable request. The JMP Pro version 16.2.0. software(SAS, Cary, NC, USA) was used for statistical analysis.
